# The genome sequence of the Spruce Carpet Moth, *Thera britannica* (Turner, 1925)

**DOI:** 10.12688/wellcomeopenres.19107.1

**Published:** 2023-03-10

**Authors:** Douglas Boyes, University of Oxford and Wytham Woods Genome Acquisition Lab, Darwin Tree of Life Barcoding collective, Wellcome Sanger Institute Tree of Life programme, Wellcome Sanger Institute Scientific Operations: DNA Pipelines collective, Tree of Life Core Informatics collective, Peter W.H. Holland, Darwin Tree of Life Consortium

**Affiliations:** 1 UK Centre for Ecology and Hydrology, Wallingford, Oxfordshire, UK; 2 University of Oxford, Oxford, Oxfordshire, UK

**Keywords:** Thera britannica, Spruce Carpet Moth, genome sequence, chromosomal, Lepidoptera

## Abstract

We present a genome assembly from an individual male *Thera britannica* (the Spruce Carpet Moth; Arthropoda; Insecta; Lepidoptera; Geometridae). The genome sequence is 381 megabases in span. Most of the assembly is scaffolded into 19 chromosomal pseudomolecules, including the assembled Z sex chromosome. The mitochondrial genome has also been assembled and is 15.9 kilobases in length. Gene annotation of this assembly on Ensembl has identified 12,457 protein coding genes.

## Species taxonomy

Eukaryota; Metazoa; Ecdysozoa; Arthropoda; Hexapoda; Insecta; Pterygota; Neoptera; Endopterygota; Lepidoptera; Glossata; Ditrysia; Geometroidea; Geometridae; Larentiinae; *Thera*; *Thera britannica* (Turner, 1925) (NCBI:txid987013).

## Background

The genus *Thera,* within the family Geometridae, contains several species of moth that are difficult to distinguish morphologically. Whether each named species is genetically distinct is controversial and much taxonomic confusion persists. *Thera britannica*, the Spruce Carpet Moth, was described by the Australian paediatrician and entomologist Alfred Jefferis Turner in 1925, and was named for an association with the British Isles. Despite its specific name, the moth is not endemic to Britain but is found widely across northern and central Europe where its larval food plants grow, primarily spruce *Picea* sp. and Douglas fir *Pseudotsuga menziesii* (UKMoths, no date; GBIF Secretariat, 2022). In the UK, the moth has two generations per year and is found throughout England, Wales, Northern Ireland and southern counties of Scotland. In Ireland, records are primarily from the south of the country. The number of records of *T. britannica* has increased hugely since 1970, presumably due to commercial planting of conifers providing new habitats for breeding ( Randle *et al.*, 2019).

The forewings of *T. britannica* are a grey-brown ground colour, traversed by a wide, deeply fluted median band of darker brown outlined in cream. Almost identical markings occur in another UK species, the grey pine carpet *T. obeliscata*, with subtle and inconsistent differences in colour; however, *T. obeliscata* has a distinct larval food plant, pine ( *Pinus* sp.). It has been suggested that the shape of each antennal segment in males is a more reliable character to distinguish between the two species ( Townsend *et al.*, 2010). To further complicate matters, a third species *T. variata* is described from mainland Europe, and sometimes considered conspecific to *T. britannica* ( South, 1961; Townsend *et al.*, 2010). Preliminary analysis of CO1 DNA barcode data does not reveal clear monophyletic groups for these three moths, suggesting either that they diverged very recently from each other, or there is occasional interbreeding, or even that they are not distinct species (P.O. Mulhair & P.W.H.H. analysis at ( BOLD, 2023)). In contrast, this analysis shows clearer distinction for *T. juniperata*, *T. fermata*, *T. cognata* and *T. cupressata*. The genome sequence reported here is from an individual with a CO1 DNA barcode closest to several previously reported *T. britannica* specimens.

A genome sequence for *T. britannica* will be useful finding variable genetic loci of use in resolving the taxonomy of this genus, and also for probing the genetic basis of adaptation to coniferous food plants.

### Genome sequence report

**Figure 1.**
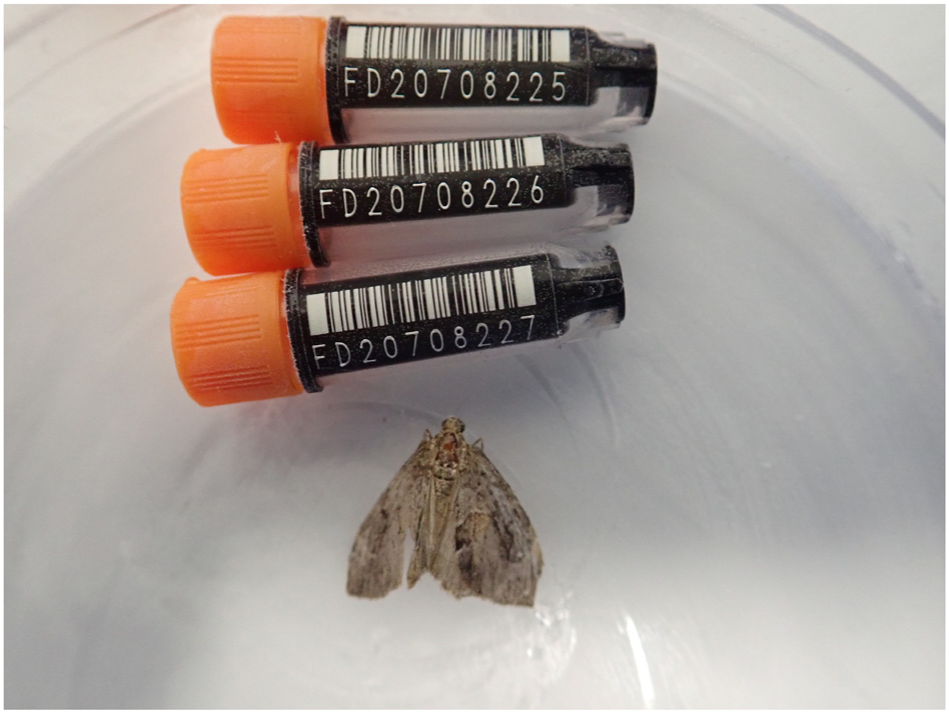
Photograph of the *Thera britannica* (ilTheBrit1) specimen used for genome sequencing.

The genome was sequenced from one male *T. britannica* specimen ( Figure 1) collected in Wytham Woods, UK (latitude 51.77, longitude –1.33). A total of 55-fold coverage in Pacific Biosciences single-molecule HiFi long reads and 117-fold coverage in 10X Genomics read clouds was generated. Primary assembly contigs were scaffolded with chromosome conformation Hi-C data. Manual assembly curation corrected 29 missing or mis-joins, and removed two haplotypic duplications, reducing the scaffold number by 4%.

**Figure 2.**
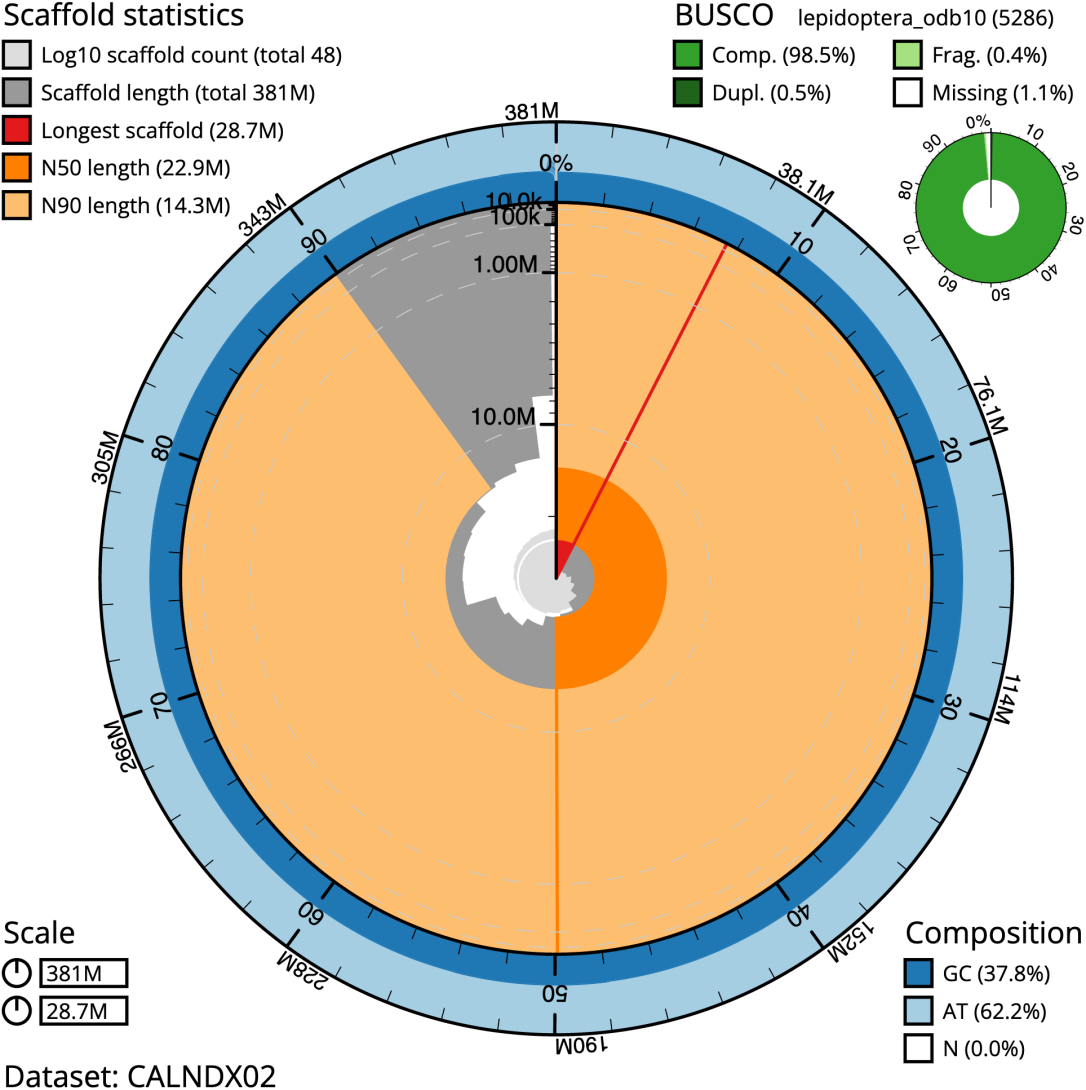
Genome assembly of *Thera britannica*, ilTheBrit1.2: metrics. The BlobToolKit Snailplot shows N50 metrics and BUSCO gene completeness. The main plot is divided into 1,000 size-ordered bins around the circumference with each bin representing 0.1% of the 380,638,026 bp assembly. The distribution of scaffold lengths is shown in dark grey with the plot radius scaled to the longest scaffold present in the assembly (28,669,802 bp, shown in red). Orange and pale-orange arcs show the N50 and N90 scaffold lengths (22,904,759 and 14,258,099 bp), respectively. The pale grey spiral shows the cumulative scaffold count on a log scale with white scale lines showing successive orders of magnitude. The blue and pale-blue area around the outside of the plot shows the distribution of GC, AT and N percentages in the same bins as the inner plot. A summary of complete, fragmented, duplicated and missing BUSCO genes in the lepidoptera_odb10 set is shown in the top right .An interactive version of this figure is available at https://blobtoolkit.genomehubs.org/view/ilTheBrit1.2/dataset/CALNDX02/snail.

The final assembly has a total length of 380.6 Mb in 48 sequence scaffolds with a scaffold N50 of 22.9 Mb ( Table 1). Most (99.71%) of the assembly sequence was assigned to 19 chromosomal-level scaffolds, representing 18 autosomes and the Z sex chromosome. Chromosome-scale scaffolds confirmed by the Hi-C data have been named in order of size ( Figure 2– Figure 5; Table 2). The assembly has a BUSCO v5.3.2 ( Manni *et al.*, 2021) completeness of 98.5% (single 98.1%, duplicated 0.5%) using the lepidoptera_odb10 reference set. While not fully phased, the assembly deposited is of one haplotype. Contigs corresponding to the second haplotype have also been deposited.

**Table 1.** Genome data for *Thera britannica*, ilTheBrit1.2.

Project accession data		
Assembly identifier	ilTheBrit1.2	
Species	*Thera britannica*	
Specimen	ilTheBrit1	
NCBI taxonomy ID	987013	
BioProject	PRJEB52209	
BioSample ID	SAMEA8603178	
Isolate information	male, ilTheBrit1, thorax (PacBio and 10X); abdomen (RNA-Seq), head (Hi-C)	
Assembly metrics *		*Benchmark*
Consensus quality (QV)	59.5	*≥ 50*
*k*-mer completeness	100%	*≥ 95%*
BUSCO **	C:98.5%[S:98.1%,D:0.5%], F:0.4%,M:1.1%,n:5,286	*C ≥ 95%*
Percentage of assembly mapped to chromosomes	99.71%	*≥ 95%*
Sex chromosomes	Z chromosome	*localised homologous pairs*
Organelles	Mitochondrial genome assembled	*complete single alleles*
Raw data accessions		
PacificBiosciences SEQUEL II	ERR9630943, ERR9630944	
10X Genomics Illumina	ERR9580474–ERR9580477	
Hi-C Illumina	ERR9580479	
PolyA RNA-Seq Illumina	ERR9580478	
Genome assembly		
Assembly accession	GCA_939531255.2	
*Accession of alternate haplotype*	GCA_939531245.2	
Span (Mb)	380.6	
Number of contigs	140	
Contig N50 length (Mb)	6.4	
Number of scaffolds	48	
Scaffold N50 length (Mb)	22.9	
Longest scaffold (Mb)	28.7	
Genome annotation		
Number of protein-coding genes	12,457	
Number of non-coding genes	1,912	
Number of gene transcripts	21,718	

^*^ Assembly metric benchmarks are adapted from column VGP-2020 of “Table 1: Proposed standards and metrics for defining genome assembly quality” from ( Rhie *et al.*, 2021). ^**^ BUSCO scores based on the lepidoptera_odb10 BUSCO set using v5.3.2. C = complete [S = single copy, D = duplicated], F = fragmented, M = missing, n = number of orthologues in comparison. A full set of BUSCO scores is available at https://blobtoolkit.genomehubs.org/view/ilTheBrit1.1/dataset/CALNDX01/busco.

**Figure 3.**
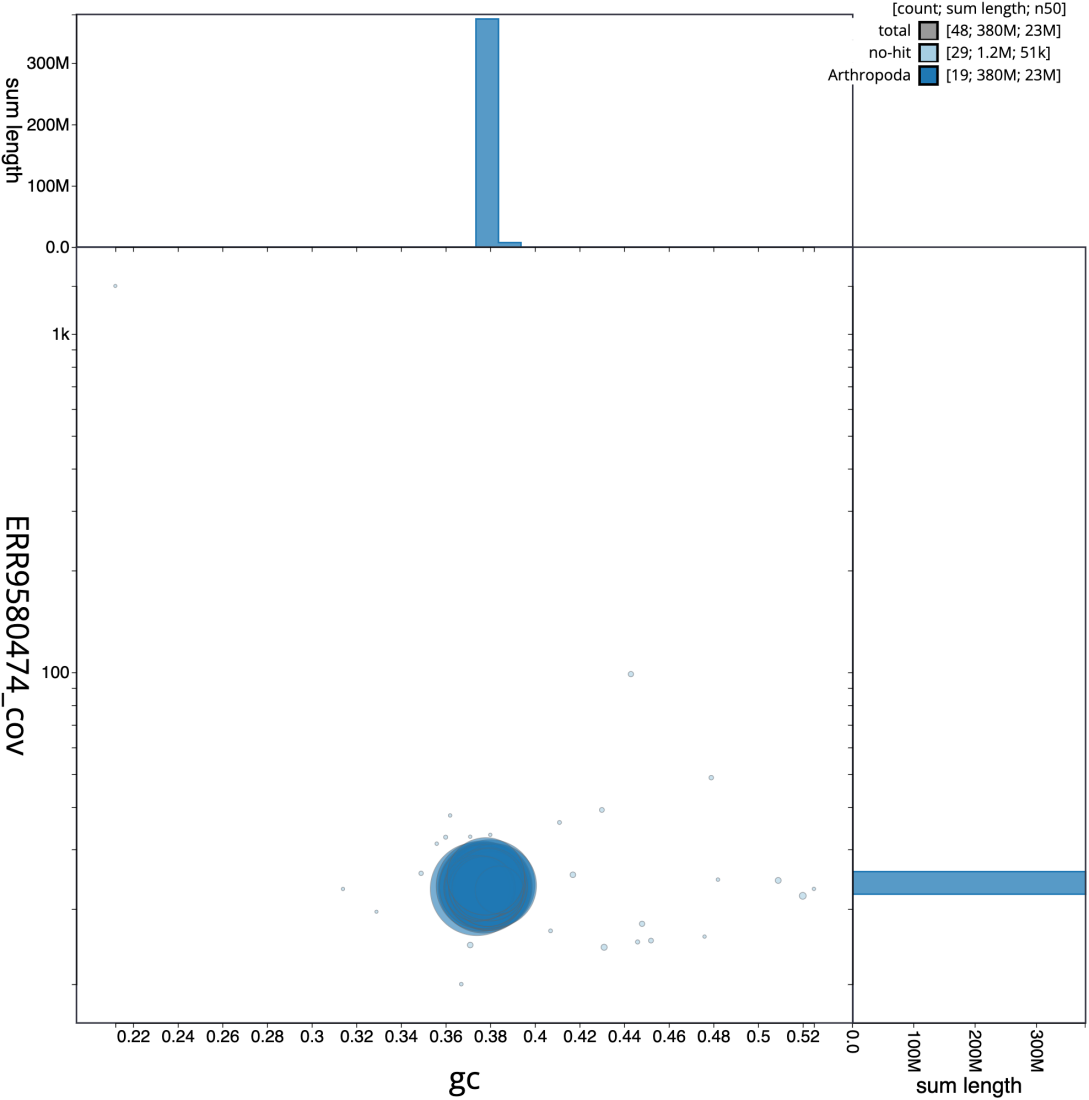
Genome assembly of *Thera britannica*, ilTheBrit1.2: GC coverage. BlobToolKit GC-coverage plot. Scaffolds are coloured by phylum. Circles are sized in proportion to scaffold length. Histograms show the distribution of scaffold length sum along each axis. An interactive version of this figure is available at https://blobtoolkit.genomehubs.org/view/ilTheBrit1.2/dataset/CALNDX02/blob.

**Figure 4.**
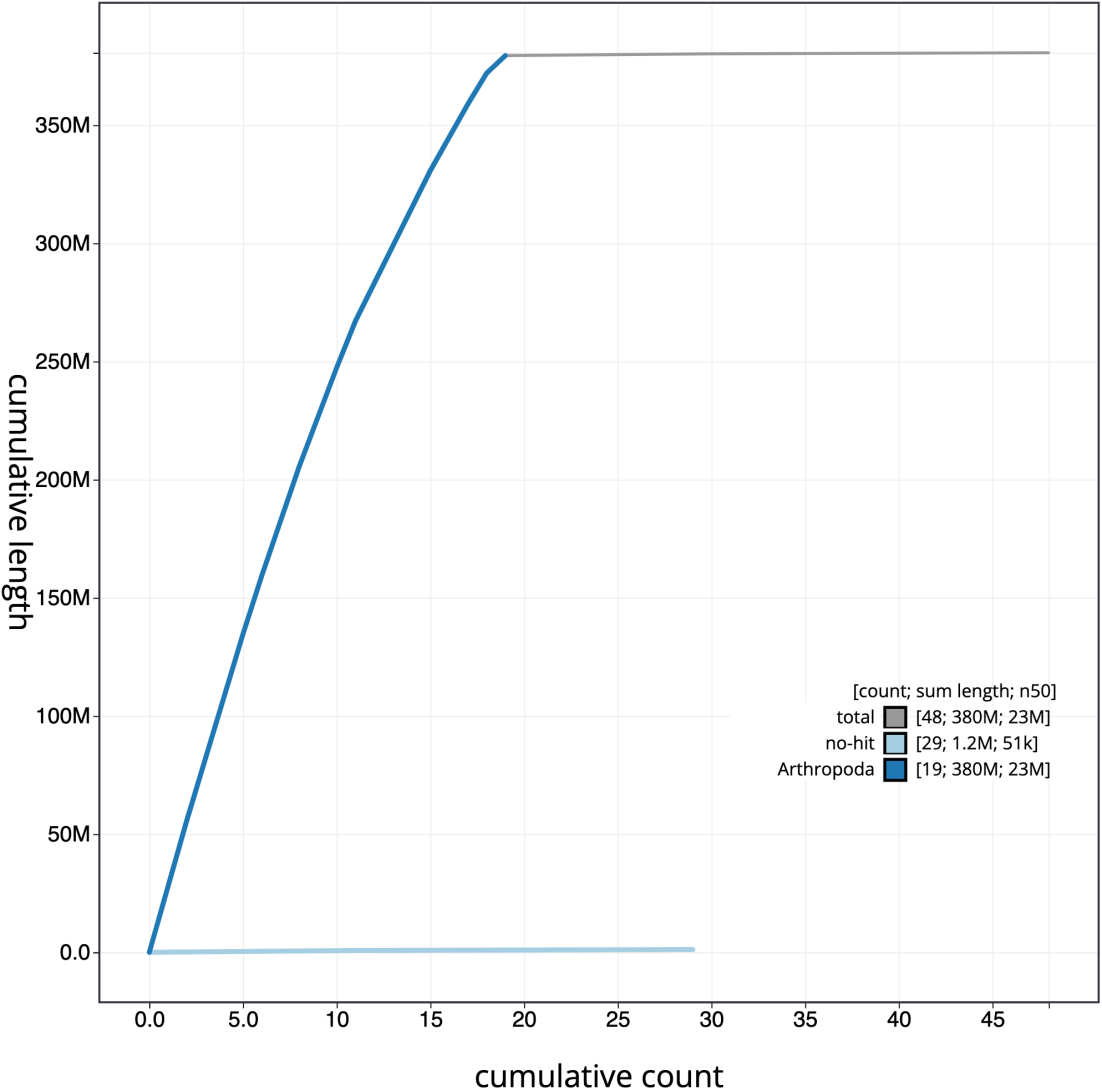
Genome assembly of *Thera britannica*, ilTheBrit1.2: cumulative sequence. BlobToolKit cumulative sequence plot. The grey line shows cumulative length for all scaffolds. Coloured lines show cumulative lengths of scaffolds assigned to each phylum using the buscogenes taxrule. An interactive version of this figure is available at https://blobtoolkit.genomehubs.org/view/ilTheBrit1.2/dataset/CALNDX02/cumulative.

**Figure 5.**
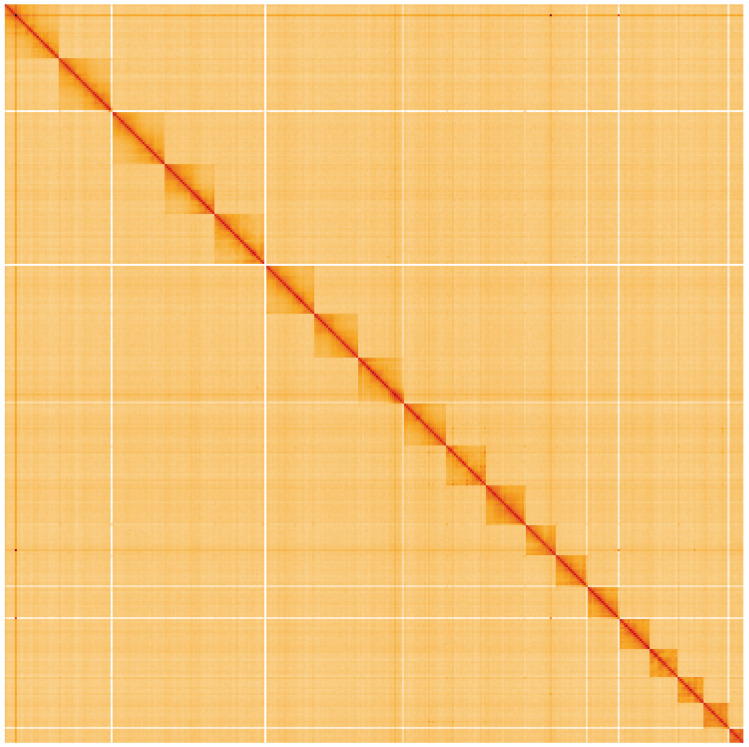
Genome assembly of *Thera britannica*, ilTheBrit1.2: Hi-C contact map. Hi-C contact map of the ilTheBrit1.2 assembly, visualised using HiGlass. Chromosomes are shown in order of size from left to right and top to bottom. An interactive version of this figure may be viewed at https://genome-note-higlass.tol.sanger.ac.uk/l/?d=R0eN8cr5RKWswnzXKKOQWQ.

**Table 2.** Chromosomal pseudomolecules in the genome assembly of *Thera britannica*, ilTheBrit1.2.

INSDC accession	Chromosome	Length	GC%
OW618034.2	1	28.67	37
OW618035.2	2	27.18	37.5
OW618036.2	3	26.94	37.5
OW618037.2	4	26.28	38
OW618038.2	5	25.70	37.5
OW618039.2	6	24.57	37.5
OW618040.2	7	23.31	37.5
OW618041.2	8	22.90	38
OW618042.2	9	21.51	37.5
OW618043.2	10	20.35	37.5
OW618045.2	11	16.11	37.5
OW618046.2	12	16.03	37.5
OW618047.2	13	15.86	37.5
OW618048.2	14	15.66	37.5
OW618049.2	15	14.26	37
OW618050.2	16	13.87	37.5
OW618051.2	17	13.05	37.5
OW618052.2	18	7.48	38
OW618044.2	Z	19.73	37.5
OW618053.2	Mitochondrion	0.02	19.5

### Genome annotation report

The *T. brittanica* genome assembly GCA_939531255.1 was annotated using the Ensembl rapid annotation pipeline ( Table 1; https://rapid.ensembl.org/Thera_britannica_GCA_939531255.1/). The resulting annotation includes 21,718 transcribed mRNAs from 12,457 protein-coding and 1,912 non-coding genes.

## Methods

### Sample acquisition and nucleic acid extraction

A male *T. britannica* specimen (ilTheBrit1) was collected in Wytham Woods, Oxfordshire (biological vice-county: Berkshire), UK (latitude 51.77, longitude –1.34) on 8 September 2020, using a light trap. The specimen was collected and identified by Douglas Boyes (University of Oxford) and snap-frozen on dry ice.

DNA was extracted at the Tree of Life laboratory, Wellcome Sanger Institute (WSI). The ilTheBrit1 sample was weighed and dissected on dry ice with tissue set aside for Hi-C sequencing. Thorax tissue was disrupted using a Nippi Powermasher fitted with a BioMasher pestle. High molecular weight (HMW) DNA was extracted using the Qiagen MagAttract HMW DNA extraction kit. Low molecular weight DNA was removed from a 20 ng aliquot of extracted DNA using 0.8X AMpure XP purification kit prior to 10X Chromium sequencing; a minimum of 50 ng DNA was submitted for 10X sequencing. HMW DNA was sheared into an average fragment size of 12–20 kb in a Megaruptor 3 system with speed setting 30. Sheared DNA was purified by solid-phase reversible immobilisation using AMPure PB beads with a 1.8X ratio of beads to sample to remove the shorter fragments and concentrate the DNA sample. The concentration of the sheared and purified DNA was assessed using a Nanodrop spectrophotometer and Qubit Fluorometer and Qubit dsDNA High Sensitivity Assay kit. Fragment size distribution was evaluated by running the sample on the FemtoPulse system.

RNA was extracted from abdomen tissue of ilTheBrit1 in the Tree of Life Laboratory at the WSI using TRIzol, according to the manufacturer’s instructions. RNA was then eluted in 50 μl RNAse-free water and its concentration assessed using a Nanodrop spectrophotometer and Qubit Fluorometer using the Qubit RNA Broad-Range (BR) Assay kit. Analysis of the integrity of the RNA was done using Agilent RNA 6000 Pico Kit and Eukaryotic Total RNA assay.

### Sequencing

Pacific Biosciences HiFi circular consensus and 10X Genomics read cloud DNA sequencing libraries were constructed according to the manufacturers’ instructions. Poly(A) RNA-Seq libraries were constructed using the NEB Ultra II RNA Library Prep kit. DNA and RNA sequencing was performed by the Scientific Operations core at the WSI on Pacific Biosciences SEQUEL II (HiFi), Illumina HiSeq 4000 (RNA-Seq) and Illumina NovaSeq 6000 (10X) instruments. Hi-C data were also generated from head tissue of ilTheBrit1 using the Arima v2 kit and sequenced on the Illumina NovaSeq 6000 instrument.

### Genome assembly

Assembly was carried out with Hifiasm ( Cheng *et al.*, 2021) and haplotypic duplication was identified and removed with purge_dups ( Guan *et al.*, 2020). One round of polishing was performed by aligning 10X Genomics read data to the assembly with Long Ranger ALIGN, calling variants with FreeBayes ( Garrison & Marth, 2012). The assembly was then scaffolded with Hi-C data ( Rao *et al.*, 2014) using YaHS ( Zhou *et al.*, 2023). The assembly was checked for as described previously ( Howe *et al.*, 2021). Manual curation was performed using HiGlass ( Kerpedjiev *et al.*, 2018) and Pretext ( Harry, 2022). The mitochondrial genome was assembled using MitoHiFi ( Uliano-Silva *et al.*, 2022), which performed annotation using MitoFinder ( Allio *et al.*, 2020). The genome was analysed, and BUSCO scores were generated within the BlobToolKit environment ( Challis *et al.*, 2020). Table 3 contains a list of all software tool versions used, where appropriate.

**Table 3.** Software tools and versions used.

Software tool	Version	Source
BlobToolKit	4.0.7	Challis *et al.*, 2020
FreeBayes	1.3.1-17-gaa2ace8	Garrison & Marth, 2012
Hifiasm	0.16.1-r375	Cheng *et al.*, 2021
HiGlass	1.11.6	Kerpedjiev *et al.*, 2018
Long Ranger ALIGN	2.2.2	https://support.10xgenomics.com/genome-exome/ software/pipelines/latest/advanced/other-pipelines
MitoHiFi	2	Uliano-Silva *et al.*, 2022
PretextView	0.2	Harry, 2022
purge_dups	1.2.3	Guan *et al.*, 2020
YaHS	yahs-1.1.91eebc2	Zhou *et al*., 2023

### Genome annotation

The Ensembl gene annotation system ( Aken *et al.*, 2016) was used to generate annotation for the *T. britannica* assembly (GCA_939531255.1). Annotation was created primarily through alignment of transcriptomic data to the genome, with gap filling via protein to-genome alignments of a select set of proteins from UniProt ( UniProt Consortium, 2019).

### Ethics and compliance issues

The materials that have contributed to this genome note have been supplied by a Darwin Tree of Life Partner. The submission of materials by a Darwin Tree of Life Partner is subject to the Darwin Tree of Life Project Sampling Code of Practice. By agreeing with and signing up to the Sampling Code of Practice, the Darwin Tree of Life Partner agrees they will meet the legal and ethical requirements and standards set out within this document in respect of all samples acquired for, and supplied to, the Darwin Tree of Life Project. All efforts are undertaken to minimise the suffering of animals used for sequencing. Each transfer of samples is further undertaken according to a Research Collaboration Agreement or Material Transfer Agreement entered into by the Darwin Tree of Life Partner, Genome Research Limited (operating as the Wellcome Sanger Institute), and in some circumstances other Darwin Tree of Life collaborators.

## Data Availability

European Nucleotide Archive: *Thera britannica* (spruce carpet). Accession number PRJEB52209; https://identifiers.org/ena.embl/PRJEB52209. ( Wellcome Sanger Institute, 2022) The genome sequence is released openly for reuse. The *Thera britannica* genome sequencing initiative is part of the Darwin Tree of Life (DToL) project. All raw sequence data and the assembly have been deposited in INSDC databases. Raw data and assembly accession identifiers are reported in Table 1.

## Author information

Members of the University of Oxford and Wytham Woods Genome Acquisition Lab are listed here: https://doi.org/10.5281/zenodo.4789928.

Members of the Darwin Tree of Life Barcoding collective are listed here: https://doi.org/10.5281/zenodo.4893703.

Members of the Wellcome Sanger Institute Tree of Life programme are listed here: https://doi.org/10.5281/zenodo.4783585.

Members of Wellcome Sanger Institute Scientific Operations: DNA Pipelines collective are listed here: https://doi.org/10.5281/zenodo.4790455.

Members of the Tree of Life Core Informatics collective are listed here: https://doi.org/10.5281/zenodo.5013541.

Members of the Darwin Tree of Life Consortium are listed here: https://doi.org/10.5281/zenodo.4783558.
