# Supplementary material for: The genome sequence of the Spruce Carpet Moth, Thera britannica (Turner, 1925)
Source: Wellcome Open Res. 2023 Mar 10;8:114. [Version 1] doi: 10.12688/wellcomeopenres.19107.1 (PMC10318373; doi:10.12688/wellcomeopenres.19107.1)
Supplement: Peer review history

# Peer review: Reviewer response for version 1

**DOI:** 10.21956/wellcomeopenres.21183.r59570

**Published:** 2023-07-03

**Authors:** Jerome H. L. Hui

**Affiliations:** 1 School of Life Sciences, The Chinese University of Hong Kong, Hong Kong, China

Boyes and colleagues report the genome sequence of spruce carpet moth *Thera britannica*. This species can be found throughout Britain. As written in the background, there could be difficulties in distinguishing this species with other moths in the same genus; therefore, this new genome resource will be very useful for further studies, ranging from identifying cryptic species, revealing their population structures, dissecting host plant-insect relationships, to understanding their evolution.

 This genome resource is excellent from the summary statistics, with high BUSCO numbers, high sequence continuity, and majority of sequences contained on the 19 pseudochromosomes (plus mitochondrion). All in all, this is a valuable contribution.

Are sufficient details of methods and materials provided to allow replication by others?

Yes

Is the rationale for creating the dataset(s) clearly described?

Yes

Are the datasets clearly presented in a useable and accessible format?

Yes

Are the protocols appropriate and is the work technically sound?

Yes

Reviewer Expertise:

Genomics, evolution, invertebrates

I confirm that I have read this submission and believe that I have an appropriate level of expertise to confirm that it is of an acceptable scientific standard.

# Peer review: Reviewer response for version 1

**DOI:** 10.21956/wellcomeopenres.21183.r55546

**Published:** 2023-03-28

**Authors:** Sean T. S. Law

**Affiliations:** 1 School of Life Sciences, Simon F.S. Li Marine Science Laboratory, State Key Laboratory of Agrobiotechnology, The Chinese University of Hong Kong, Hong Kong, China

This article reports the genome assembly of a male Spruce Carpet Moth, *Thera britannica*. PacBio HiFi reads, 10X Genomics linked-reads and Hi-C data were used to assemble the genome of ~381 Mb in size, with most portions scaffolded into 19 chromosomes. The BUSCO score and N50 statistics are ideal. This assembly provides high quality genomic resource for further investigations in the evolution of lepidopterans, including the genetic structure of this species and its close relatives.

Are sufficient details of methods and materials provided to allow replication by others?

Yes

Is the rationale for creating the dataset(s) clearly described?

Yes

Are the datasets clearly presented in a useable and accessible format?

Yes

Are the protocols appropriate and is the work technically sound?

Yes

Reviewer Expertise:

Evolutionary Biology, Population genomics

I confirm that I have read this submission and believe that I have an appropriate level of expertise to confirm that it is of an acceptable scientific standard.
